# The Gluten Free Diet: Assessing Adherence in a Pediatric Celiac Disease Population

**DOI:** 10.1093/jcag/gwy067

**Published:** 2018-12-12

**Authors:** Jenna K Dowhaniuk, Heather Mileski, Joanne Saab, Perri Tutelman, Lehana Thabane, Herbert Brill

**Affiliations:** 1 Department of Pediatrics, McMaster University, Hamilton, Ontario, Canada; 2 McMaster Children’s Hospital, Hamilton Health Sciences Ontario, Canada; 3 Department of Health Research Methods, Evidence and Impact, McMaster University, Hamilton, Ontario, Canada

**Keywords:** Adherence, Celiac disease, Children, Dietitian

## Abstract

**Background:**

A strict, lifelong, gluten-free diet (GFD) remains the sole treatment for celiac disease (CD). The assessment of adherence to the GFD in pediatric studies is often based on self-report and visual analogue scales which lack proven validity. We sought to compare parental-report of GFD adherence to expert registered dietitian (RD) assessments, the best available standard.

**Methods:**

Parents of children with biopsy-proven CD scored their adherence to the GFD on a five-point Likert scale similar to that used in previous pediatric CD studies. Each family was then evaluated by an RD expert in CD management who conducted a comprehensive and standardized assessment and scored the family’s adherence. The agreement between parents and the RD was assessed using paired t test and intraclass correlation coefficient (ICC) based on their scores.

**Results:**

One hundred twenty-two children and their families participated in the study, with a median of 32 months on a GFD. Excellent adherence (score 5 out of 5) was attributed to 60.5% of the sample by the RD. The parents scored adherence higher than the RD by an average difference of 0.41 scale points (95% CI, 0.28–0.54; *P* < 0.001). The agreement between parents and the registered dietitian was poor (ICC = 0.21).

**Conclusion:**

Reliance on self-report through Likert scales for GFD adherence overestimates adherence and misses opportunities for patient and family education. Approximately 40% of children with CD have ongoing gluten exposure, highlighting the need for regular assessment by an RD expert in the GFD to identify education and counselling needs for children with CD.

Celiac disease (CD) is an immune-mediated disease of the small intestine characterized by histologic changes including lymphocytic infiltration and villous architectural changes in genetically susceptible individuals and is triggered by the consumption of gluten (1–3). Celiac disease is one of the most common chronic diseases in children, with an estimated incidence in children by age 5 as high as one in 104 in the United States (4). A strict lifelong, gluten-free diet (GFD) devoid of wheat, rye and barley remains the sole treatment for CD (1). This diet can be exceedingly restrictive and challenging for families, and many have difficulty with the chronic nature of both the disease and the GFD (5–7).

As the GFD is currently the central feature in the management of CD, it is crucial to evaluate a child’s adherence to the diet to reduce future disease complications (8). Clinicians often consider the adherence to the GFD a surrogate to the extent of disease activity of the small intestine (8).

At present, no evaluative tool exists for the evaluation of children on a GFD; therefore, there is significant variability in how adherence is assessed for children with CD (9). The North American Society for Pediatric Gastroenterology, Hepatology and Nutrition Celiac Disease Guidelines state, “There is little evidence on the most effective means of monitoring patients with CD” (1). Studies of children with CD have relied on a variety of assessments of GFD adherence including interview, dietary recall, visual analogue scales and repeat Celiac serology such as tissue transglutaminase (TTG) antibodies (9). This has led to significant variation in the adherence rates reported in children, ranging from 30% to 95% [1, 5, 9]. Reliance on serology alone may miss ongoing gluten exposure and the opportunity for intervention for children with CD (9). Recent reports of repeat endoscopy in children with CD identify that up to 19% have ongoing enteropathy despite following a GFD. Most concerning, the use of immunoglobulin A (IgA)–TTG was a poor predictor of ongoing enteropathy with a positive predictive value (PPV) of 25% (10).

The aim of this study was to determine the accuracy of self-reporting of GFD adherence. We completed a single-centre, prospective cohort study of children with biopsy-confirmed CD. We sought to compare parental-report of GFD adherence on interval scales with expert registered dietitian (RD) assessments because standardized assessment by an RD is considered the most objective noninvasive method to assess dietary adherence to a GFD (8,11).

## METHODS

### Study Population

A convenience sample of participants 18 years of age and younger with biopsy-proven CD was approached at their annual CD clinic appointment at McMaster Children’s Hospital during the 12-month study period from January 2013 to January 2014. Informed consent was obtained from all participants.

### Data Collection

The parents were asked to rank their child’s GFD adherence using a Likert scale from one to five. Nonjudgmental prompts for scores were provided. A score of one was subjectively defined as ‘we find it very difficult to follow a GFD and are unable to do so most of the time’. The prompt for score five was defined as ‘we always follow a strict gluten free diet’. If more than one parent was present, study packages were completed together. Children who were able to read independently, as determined by their parents, also scored their adherence using a similar five-point score. Reasons for any child who was unable to complete the study were documented.

Patients and their families underwent a detailed clinical evaluation by an RD expert in the management of pediatric CD for nine years. To ensure standardization, a dietary-history template, approved by an interdisciplinary team of gastroenterologists, nurses and RDs at McMaster Children’s Hospital, was implemented at each visit. The template included questions regarding hidden sources of gluten and assessed the frequency of purposeful gluten consumption. Each meeting evaluated the challenges of following a strict GFD including steps taken during travel, avoidance of cross-contamination and label reading. Eating at restaurants was thoroughly explored. All patients completed a detailed 24-hour dietary recall to further quantify GFD adherence.

Following the thorough evaluation, the RD scored each child’s GFD adherence on the same Likert scale of one to five (one = poor). To assess reliability of RD scoring, a second RD in the division of pediatric gastroenterology, also experienced with pediatric CD and the GFD, reviewed all documentation while blinded to the original score and scored each child on the same Likert scale of one to five.

The patient demographics and disease factors, including symptomatology, perceived GFD education provided and GFD understanding, were collected. The study was approved by the Research Ethics Board at McMaster University, Hamilton Ontario.

### Statistical Analysis

The primary outcome measure was defined as the difference in Likert scales between the parent and the RD. To assess if any difference existed between scores and to assess its magnitude and significance, a paired t test was used. Note that the differences are on the scale from −5 to +5, providing sufficient variability to justify the assumption of normality distribution on the difference. However, we examined the distribution and found no evidence of the normality assumption. The sample size was calculated based on a paired t test with the assumption of a clinically relevant Likert scale difference of one, SD of two, alpha = 0.05, and power 80%. The calculated sample size was 113.

In addition, the agreement between the parent and dietitian was reported as an intraclass correlation coefficient (ICC). Secondary analysis included comparison of scores (1) between the child and parent and (2) between the child and RD. To assess inter-rater reliability, the scores of both RDs were assessed as secondary outcomes.

To examine the potential factors affecting the differences of adherence scores between the parents and RD, we constructed a multiple regression model. The multiple regression model was constructed by identifying the potential factors or covariates through patient demographics and prognostic information using univariate analysis; then the variables with P ≤ 0.2 in univariate analysis were put into the multiple regression model. The variables with *P* < 0.10 remained in the final regression model. For these analyses, we used case deletion for missing data. The result of the final multiple regression model was reported as the difference and the corresponding 95% confidence interval (CI) and P value.

Patient characteristics and demographic information were analyzed descriptively. The continuous data were reported as median (minimum, maximum), and categorical data were reported as frequency and percentage (%). All analyses were conducted using STATA 13 (College Station, TX: StataCorp LP).

## RESULTS

### Sample Characteristics

**Figure 1.**
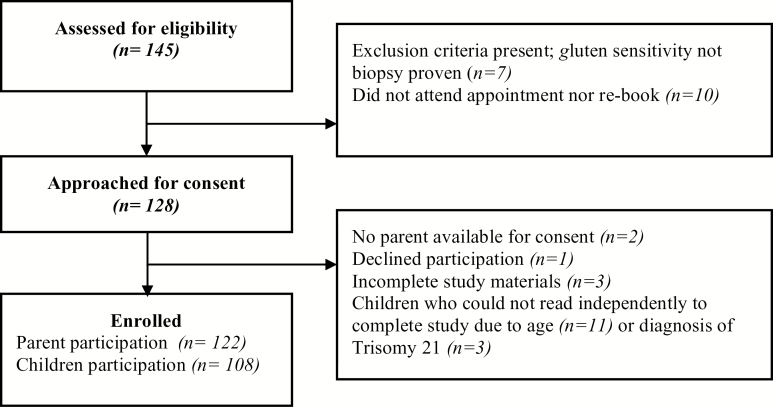
Enrollment flow chart for study

One hundred forty-five children were scheduled for clinic appointments during our study period. One family declined involvement, two parents were unavailable for consent, and three families did not complete the study packages (Figure 1). Seven children were excluded because they did not have biopsy-proven CD, and 10 did not attend their appointment nor rebook during the study period. In total, 122 parents completed the study and were included in our primary analysis. The 108 children who could read independently also completed the study. In the instances when children did not complete their own score, this was due to age (n = 11) or a diagnosis of Trisomy 21 (n = 3).

Patient characteristics are summarized in Table 1. The sample majority was female (63.9%), with a median age at diagnosis of 7.0 years (range nine months to 16 years). Median age at enrollment was 11 years (range one to 18 years). The median number of months since the start of the GFD was 32, with the maximum of 208 months. Sixty-four percent of the sample reported being moderately to severely symptomatic at the time at their diagnosis, and 68.9% reported developing symptoms if they ingested gluten. A large proportion of families received educational resources at diagnosis (94.3%). The majority of families described themselves as able to identify gluten-free foods (97.5%); however, up to 45% of parents also identified themselves as wanting additional education on the GFD. The most prevalent groups associated with CD in our sample included those with Type 1 diabetes (13.9%) and those with first-degree relatives with CD (41.3%). A limited proportion of the children and family in this sample were registered with the Canadian Celiac Association (31%). Secondary analysis evaluated the 108 children who independently completed a Likert scale of their adherence. Of this group, the median child age was 11 years old, with a range of six to 18 years.

**Table 1.** Demographic characteristics: *n* = 122

Variable	Statistics
Age, y, median (minimum, maximum)	11 (1, 18)
Gender, n (%)	
Male	44 (36.1)
Female	78 (63.9)
Age diagnosed with CD, y, median (minimum, maximum)	7.0 (0.75, 16)
Time since start of the GFD, months, median (minimum, maximum)	32 (3, 208)
Perceived severity of diagnosis, n (%)	
None	21 (17.2)
Mild	22 (18.0)
Moderate	38 (31.2)
Severe	41 (33.6)
Symptom onset prior to diagnosis, n, (%)	
Less than one year	56 (46.3)
1–3 years	34 (28.1)
Over 3 years	23 (19.7)
No symptoms/NA	8 (6.6)
Co-morbidities, n, (%)	
Autoimmune Thyroid Disease	5 (4.1)
Type 1 Diabetes	17 (13.9)
Down Syndrome	3 (2.5)
Selective IgA Deficiency	1 (0.8)
Reported Current total family income, n, (%)	
<$29,252	2 (1.7)
$29,252–$49,999	3 (2.6)
$50,000–$69,999	9 (7.7)
$70,000–$89,999	14 (12.0)
Over $ 90,000	89 (76.1)
Member of Canadian Celiac Association, n, (%)	38 (31.4)
Education resources were provided at diagnosis, n, (%)	115 (94.3)
Family member with CD diagnosis, n, (%)	50 (41.3)
Ability to identify gluten-free food items, n, (%)	119 (97.5)
Requests more education on the gluten-free diet, n, (%)	55 (45.1)
Symptoms experienced if gluten is ingested, n, (%)	
No	33 (8.0)
Yes	81 (68.6)
Unknown	4 (3.4)

Abbreviations: SD, Standard Deviation

### Adherence Scores

**Figure 2.**
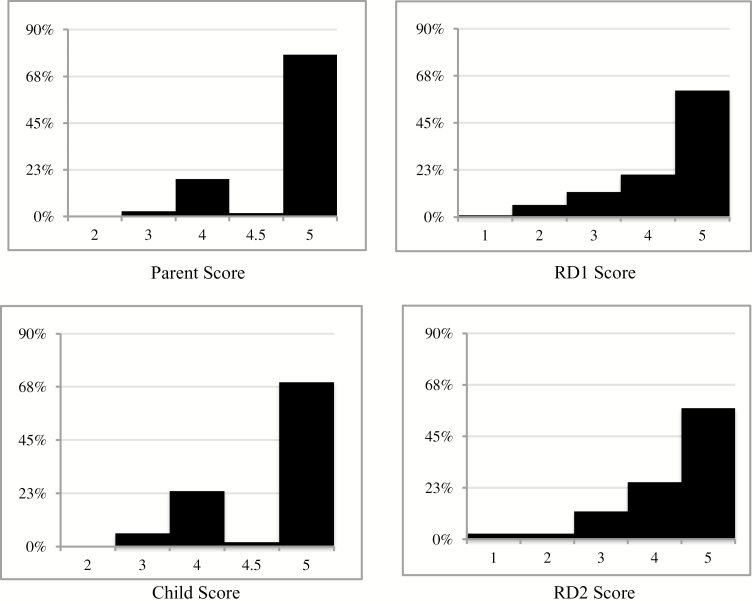
Adherence to the gluten-free diet Likert scale distribution by group. Abbreviations: RD1, registered dietitian assessing primary outcome; RD2, registered dietitian reviewing documentation

Of the 122 parents, 77.9% reported strict adherence for their children as deemed by a score of five (Figure 2). In comparison, 69.4% of children evaluated their adherence as strict by a score five. In contrast, the registered dietitian scored strict adherence (score 5) in 60.5% of the sample.

Paired t test results identified a statistically significant 0.41 Likert scale point difference (95% CI, 0.28, 0.54; *P* < 0.001) between parents and the expert RD (Table 2). The assessed agreement between groups was poor, with an ICC of 0.21. The interpretation of the ICC value is depicted in Table 2. Multiple regression identified two variables of significance (Table 3). Families who requested more information on the GFD were associated with a difference of 0. 32 Likert points between RD and parental scores (*P* < 0.017). However, children who experienced over three years of symptoms before their diagnosis were associated with an improved agreement in parental scores to RD scores (*P* < 0.037). Parental education, combined family income and the perceived ability to identify if a food is gluten-free were not identified as significant predictors.

**Table 2.** Agreements of Likert scores between groups for the evaluation of GFD adherence

Outcome	Analysis Method	Results
Parent to RD1 Scores (n = 122)	Intraclass Correlation Coefficient	0.21
	Difference in scores (95% CI), P-value	0.41 (0.28, 0.54), <0.001
Parent to Child Scores (n = 108)	Intraclass Correlation Coefficient	0.64
	Difference in scores (95% CI), P-value	0.13 (0.05, 0.20), <0.001
Child to RD1 Scores (n = 108)	Intraclass Correlation Coefficient	0.34
	Difference in scores (95% CI), P-value	0.28 (0.16, 0.39), <0.001
RD1 to RD2 Scores (n = 122)	Intraclass Correlation Coefficient	0.71
	Difference in scores (95% CI), P-value	−0.004 (−0.08, 0.07), 0.912
	Kappa Statistic	0.77

Abbreviations: RD1, registered dietitan assessing primary outcome; RD2, registered dietitian reviewing documentation. Intraclass correlation coefficient and Kappa reference values: κ < 0.2: Poor, κ 0.21–0.40: Fair, κ 0.41–0.60 Moderate, κ 0.61–0.80: Substantial, κ 0.81–1.00: Almost Perfect (19)

**Table 3.** Multiple regression analysis of paired differences in parent scores compared with dietitian scores

Variable	β coefficient (95% CI)	P value
Identified as wanting more information on the Gluten-free diet	0.32 (0. 06, 0.59)	0.017
Years of symptom prior to diagnosis		
1–3 years	−0.30 (−0.62, 0.02)	0.066
Over 3 years	−0.37 (−0.74, −0.02)	0.037
Perceived ability to identify if a food is gluten free	−0.74 (−1.54, 0.06)	0.070
Any negative symptom if child ingest gluten	−0.24 (−0.49, 0.02)	0.070

Abbreviations: *β*, beta

Child scores compared with the RD scores had an ICC of 0.34—better than parents but still fairly weak. The Likert scale point difference was 0.28 (95% CI, 0.16, 0.39, *P* < 0.001). Children and parents had moderate agreement with an ICC of 0.64. Inter-rater reliability scores by the two RDs had an ICC of 0.71, signifying substantial agreement.

## Discussion

The current study highlights the additional information that can be obtained from RD evaluation when assessing GFD adherence. Registered dietician experts in CD offer a unique opportunity to further assess the complexity of adherence to the GFD and provide immediate intervention and education (12–14). Multiple CD guidelines stress the importance of the RD in CD monitoring (1,13). In addition, regular follow-up with an RD has been correlated with improved adherence and highlighted as important to families (14–17).

This study provides further evidence of the poor correlation between self-reported adherence and a comprehensive dietitian assessment. Although the study did not identify a clinically significant difference of one Likert point, a statistically significant difference was identified between parents and dietitian scores, highlighting that analogue scales are a suboptimal method to measure adherence. Our study has provided further evidence of the limitations and overestimation of self-report, thus highlighting the missed opportunity for further education and intervention if relying on simple interval scales.

Self-report scores of adherence may be subject to reporting biases, lack objectivity, and often provide insufficient detail (9). A previous literature review of GFD adherence in children identified a lack of standardization for assessment of the GFD (9). Of 21 studies included in analysis, nine focused on a unimodal adherence assessment approach (9). Three of these identified adherence to the GFD as their primary outcome, and their methods for assessment were based solely on self-report (9). Due to lack of standardization of assessments in clinical research, the generalizability of this data remains limited, and the degree of heterogeneity precludes meta-analysis (9). Our study provides an adherence estimate of 60.5% based on a comprehensive RD evaluation.

Gluten-free diet comprehension remains a limiting factor for a family’s strict adherence. While 94% of our families received education at diagnosis, a large percentage of parents in this cohort self-identify as wanting further education on the GFD (45.1%). As only 60.5% of children in this cohort are strictly adherent to the GFD, this study provides evidence that further education and intervention would be useful during follow-up appointments. It is clear that education and knowledge of the GFD remain an important domain in GFD adherence and can be a focus for further intervention.

The challenges of adhering to a GFD can be complex and encompass a lack of knowledge of the GFD, gluten contamination, labeling discrepancies and difficulty with the restrictive nature of the diet (5–18). As with many chronic medical diagnoses, self-report is also subject to social desirability bias, especially when evaluated by one’s medical team (9). It is well described that GFD dietary transgressions in adults are often unintentional and that diet comprehension partially explains discrepancy between expert and self-assessment (8). The difference in scores between parents and RDs in our study may be secondary to the RD’s ability to identify sources of gluten in the child’s diet of which the family was unaware.

There are several limitations to this study. First, subjects were recruited from a single centre. The recruitment of children from clinic may result in overestimation of adherence given their dedication to follow-up and interest in partaking in research studies. The families who did not book follow-up for missed appointments may well have lower adherence rates, resulting in an overestimate of overall adherence. While clinical interviews can be subject to a lack of standardization, our study utilized a single RD assessment during the entire 12-months and improved reliability with a second RD’s assessment. To compare data between groups, the study design required the assignment of arbitrary cutoffs for GFD adherence (one through five). While definitions should be related to disease-specific outcomes, this information is unknown at this time. We have also used the t test to compare the scores between the groups, which assumes that the differences in ordinal scores are continuous (19). Furthermore, missing data were ignored for multivariable analyses which may be a limitation.

In conclusion, children with celiac disease and their parents overestimate adherence to a GFD when compared with formal assessment by an RD. Most children and families are adherent to the GFD; however, up to 40% can be identified by an RD as having ongoing gluten exposures. Further research is required to develop and validate pediatric-focused assessments of GFD adherence with a focus on the child with small dietary transgressions that may not be identified with bioassays, self-report or simple analogue scales (9,10). Assessment based on self-report requires standardization and validation if it is to be used as an outcome measure in clinical studies on CD. In the interim, regular evaluations by an RD expert in the GFD in clinical practice will identify children with ongoing gluten exposures.
